# Effectiveness of a Smartphone App (BioBase) for Reducing Anxiety and Increasing Mental Well-Being: Pilot Feasibility and Acceptability Study

**DOI:** 10.2196/18067

**Published:** 2020-11-10

**Authors:** Jamie M Kawadler, Nicola Rose Hemmings, Sonia Ponzo, Davide Morelli, Geoffrey Bird, David Plans

**Affiliations:** 1 Huma Therapeutics Limited 13th Floor Millbank Tower, 21-24 Millbank London United Kingdom; 2 Department of Engineering Science Institute of Biomedical Engineering University of Oxford Oxford United Kingdom; 3 Department of Experimental Psychology University of Oxford Oxford United Kingdom; 4 Department of SITE University of Exeter Exeter United Kingdom

**Keywords:** health and well-being, health promotion, organizational and leadership support, workplace

## Abstract

**Background:**

The prevalence of workplace-related stress and anxiety is high, resulting in stress-related physical and mental illness. Digital self-guided interventions aimed at key areas of workplace design may be able to provide remote anxiolytic effects.

**Objective:**

The aim of this feasibility study is to assess changes in anxiety and mental well-being after use of the BioBase programme, a mobile phone platform for psycho-educational modules, tools, and real-time feedback of physiological data.

**Methods:**

A 4-week observational study was carried out in 55 healthy adults who were screened for stress with the Depression Anxiety Stress Scale (DASS) Stress subscale. Participants completed anxiety (6-item State-Trait Anxiety Inventory [STAI]) and mental well-being (Warwick-Edinburgh Mental Well-being Scale [WEMWBS]) questionnaires at baseline and at 4 weeks. Feedback questionnaires were administered after 4 weeks.

**Results:**

After 4 weeks of using the programme and controlling for any effect of being paid to take part in the study, STAI significantly decreased (baseline mean 45.52 [SD 13.2]; 4-week mean 39.82 [SD 11.2]; *t*_54_=–3.51; *P*<.001; CI –8.88 to –2.52; Cohen *d*=0.96) and WEMWBS significantly increased (baseline mean 48.12 [SD 6.4]; 4-week mean 50.4 [SD 6.9]; *t*_53_=2.41; *P*=.019; CI 0.44-4.23; Cohen *d*=0.66). Further, higher baseline stress was significantly associated with a greater decrease in STAI (*t*_53_=–3.41; *P*=.001; CI –8.10 to –2.10; *R*^2^=0.180) and a greater increase in WEMWBS (*t*_52_=2.41; *P*=.019; CI 0.38-4.11, *R*^2^=0.101). On feedback, participants found the programme easy to use/navigate, with the content being acceptable and relevant to workplace-related stressors; 70% (21/30) of participants would recommend the programme to a friend.

**Conclusions:**

The BioBase programme is a potentially effective intervention in decreasing anxiety and increasing mental well-being, with larger changes in those with higher baseline levels of stress.

## Introduction

The workplace can be a major cause of anxiety that leads to depression and burnout [1-4] as well as stress-related illnesses [5,6]. In the UK, 12.8 million working days were lost in 2018-2019 due to mental ill health, equating to 21.1 days lost per year per working individual [7].

Interventions in the workplace may be able to reduce the effects of anxiety or facilitate the recovery of employees with anxiety [1]. Structured and targeted psychological interventions have been found to be more effective than general stress management training [8,9], and those employees experiencing higher levels of anxiety have been shown to benefit most [10]. Smartphone apps offer a practical, scalable, accessible, and cost-effective solution to promoting employee mental health [11-13]. As smartphone use becomes more ubiquitous, a mobile solution to mental health is becoming increasingly more common [13-15].

Many therapist-assisted or guided workplace digital interventions have been shown to be effective in reducing work-related stress [12,16-22]; however, these suffer from the limited availability and high cost of more traditional forms of support, and therefore are rarely available to the entirety of the workforce [9]. Self-guided digital interventions, which overcome some of these barriers for individuals seeking support, have also shown promising results in reducing stress and anxiety [9,23,24].

Engaging, innovative, and effective interventions that address specific workplace-related mental health difficulties are still needed. Integrated technology in self-guided interventions offers several advantages that increase engagement, behavior change, and positive psychological outcomes [25], such as personalized mental health/well-being summaries and insights [1], real-time monitoring of health data from linked biosensors [20,26], and a secure and private means to access confidential support [14,27]. Combined interventions that utilize advanced technology for real-time data feedback plus active therapeutic content may yield a synergistic effect to decrease anxious symptoms, especially in those with higher baseline levels of stress and anxiety [10].

The BioBase programme (by BioBeats Ltd) includes a mobile app, BioBase, used in conjunction with its wrist-worn wearable BioBeam. The programme offers therapeutic content in a modular format in addition to tools such as deep breathing exercises and mood tracking; these data are integrated with passively collected data on activity, heart rate, and sleep duration and quality that are presented to the users via a dashboard view. This information is used to inform individuals off their current well-being, and then to direct the individual to the most appropriate intervention modules. As yet, the effectiveness of this type of self-guided combined intervention has not yet been investigated in working individuals.

The aim of this study is to evaluate the usability, acceptability, feasibility, and preliminary efficacy of BioBase in individuals in full-time employment with varied levels of stress on perceived anxiety and mental well-being. We hypothesize that after 4 weeks of using the programme (1) there will be a significant decrease in anxiety and (2) a significant increase in mental well-being compared to baseline. Further, we hypothesize that (3) higher baseline levels of self-reported stress will be associated with larger decreases in anxiety and increases in well-being. This paper first tests these hypotheses and then describes the participants’ reported acceptability, usability, and feasibility, as well as any evoked behavior change.

## Methods

### BioBase Programme

The BioBase programme consists of the BioBase smartphone app platform that integrates data from its paired wrist-worn wearable. BioBase includes workplace-specific psycho-educational content based on the job demands-resources model [28] and combines elements of mindfulness, cognitive behavioral therapy and behavioral activation theory. The content consists of 42 modules designed around 3 courses based on the UK Health & Safety Executive’s (HSE) work stressor dimensions: demands, control, and social support. These courses highlight the importance of lifestyle factors, such as sleep awareness, sleep hygiene, and activity levels, as well as recognizing internal physiological and emotional processes as a trigger for stress. The objective of all the courses is to help the user identify and use effective coping strategies: the demands course aims to reduce the perceived stress due to overwhelming demands, the control course aims to increase a person’s perception of control to avoid burnout, and the support course aims to increase social connections and social support. Each course has 14 modules, presented in either text or audio format, taking approximately 3-5 minutes to complete and containing an action or tool to complete in-app or in the workplace. To personalize content based on the individual’s needs, individuals first complete a tailored questionnaire (HSE management standards indicator tool) and the modules pertaining to the area with the lowest score are then recommended (Multimedia Appendix 1).

Data on physical activity, sleep quality, and heart rate are collected via its paired wearable device, BioBeam, and fed back to the individual via a dashboard view in order to increase awareness of the individual’s current well-being state and to help them learn how physiological patterns are related to their well-being in parallel with the content of the modules.

The app also contains on-demand tools such as an in-built ecological momentary assessment based on the Circumplex Model of Affect [29], allowing individuals to log their mood in the moment, and reflect back on their entries at a later date to gain insights into patterns and themes, and diaphragmatic breathing exercises for stress reduction.

### Recruitment

Paid participants were individually recruited from a recruitment agency specializing in user research and usability testing (People for Research UK) alongside unpaid participants recruited through convenience sampling through a local Facebook campaign. Recruitment of small businesses was also conducted by a local small business network.

Participants were eligible to take part if they were aged between 18 and 65, in full-time employment, had access to an Apple iPhone, and were able to read and understand English. Exclusion criteria were a history of clinical diagnosis of a mental health or neurological disorder, pregnancy, or current engagement in counselling or therapy. Ethical approval was granted by the University of Exeter and informed consent was obtained electronically from all participants.

### Procedure

The study lasted 4 weeks. Participants were screened for inclusion and exclusion criteria using an online questionnaire. At baseline, online questionnaires were used to collect demographic information on age, gender, education, and employment categories, as well as self-reported stress from the Stress subscale of the 21-item Depression Anxiety Stress Scale (DASS-21) [30]. The stress subscale consists of 7 questions with range 0-21 and relates to the experience of stress symptoms in the previous week. The internal consistency of the scale is good (Cronbach α=.85) and construct validity measured in previous studies is also good [31].

At baseline and at 4 weeks, questionnaires included the 6-item State-Trait Anxiety Inventory (STAI; [32]) and the Warwick-Edinburgh Mental Well-being Scale (WEMWBS; [33]). The 6-item STAI measures state anxiety, with responses ranging from 1 (Not at all) to 4 (Very much). Scaled scores are obtained by multiplying the summed responses to each item by 20 and subsequently dividing the score by 6 (range 20-80). The STAI is widely used and has a good internal consistency (α=.92) [32].

The WEMWBS is a 14-item scale assessing subjective mental well-being. Scoring is obtained by summing each response, ranging from 1 (None of the time) to 5 (All of the time) (range 14-70). WEMWBS has been validated for use in the UK with those aged 16 and above with good internal consistency (α=.91) [33].

Participants were asked to engage naturally with the app, but encouraged to continuously wear the BioBeam, use the BioBase app for around 5 minutes per day for 4 weeks, and complete at least 14 daily 3-5-minute-long modules. Measures of app engagement consisted of number of days in which the app was opened (ie, “active” days), total duration of use, number of modules completed, and total tools completed over the 4 weeks.

An additional feedback questionnaire with multiple-choice questions and free-text answers was distributed at the end of the study along with an optional semistructured interview with the researcher to expand on their responses in the feedback form. Quotes from the free text and interviews were given to illustrate insights into the feasibility, usability, and acceptability of the BioBase programme.

### Statistical Analysis

Analysis was performed using R version 3.6.0 (R Foundation Statistical Computing) [34]. To test the first hypothesis of change in STAI and WEMWBS scores between baseline and 4 weeks, variables were first tested for a normal distribution using Shapiro–Wilk tests and linear mixed-effects models (R package: “lmer”) with participants as random effects were fitted to the data.

To test the third hypothesis that baseline DASS stress scores would be associated with greater decreases in STAI and greater increases in WEMWBS, baseline DASS stress scores were first transformed using ordered quantiles to follow a normal distribution (method: OPQ, R package: “bestNormalize”). Linear regression models were used to determine association between baseline DASS stress scores and change in STAI and WEMWBS scores. All analyses used *paid* status as a covariate. *P*-values were considered statistically significant at <.05.

## Results

### Participants

A total of 70 participants were initially recruited; 4 were excluded due to current engagement in counselling/therapy and 1 was excluded because they were employed part-time. The final sample consisted of 55 participants (25 paid and 30 unpaid) as 10 participants did not complete the exit questionnaires. While paid participants were significantly older, there were no significant differences between paid and unpaid participants in terms of gender distribution; education or employment category; or baseline stress, anxiety, or well-being scores (Table 1).

**Table 1:** Sample demographics of participants with baseline self-reported stress on DASS (N=55).

Participant demographics		Paid	Unpaid	*t* test (*df*) or chi-square (*df*)	*P* value
Total participants		25 (45)	30 (55)		
Age, mean (SD), years		38.6 (13.3)	31.4 (6.7)	2.45 (54)	.02
**Gender**				0 (54)^a^	>.99
	Female, n (%)	14 (56)	16 (53)		
	Male, n (%)	11 (44)	14 (47)		
**Education**					.35^b^
	School (age 16), n (%)	3 (12)	0 (0)		
	Sixth form/college (age 18), n (%)	3 (12)	8 (27)		
	Some university, n (%)	1 (4)	1 (3)		
	2-year degree, n (%)	4 (16)	4 (13)		
	4-year degree, n (%)	10 (40)	14 (47)		
	More than 4-year degree, n (%)	4 (16)	3 (10)		
**Job category^c^**					.43^b^
	Administrative, n (%)	5 (20)	2 (7)		
	Service, n (%)	2 (8)	1 (3)		
	Technical, n (%)	1 (4)	4 (14)		
	Sales, n (%)	2 (8)	6 (21)		
	Professional, n (%)	10 (40)	10 (34)		
	Executive, n (%)	5 (20)	6 (21)		
Baseline DASS^d^ stress, mean (SD)		13.7 (8.0)	16.7 (9.9)	1.23 (53)	.22
Baseline STAI^e^, mean (SD)		43.9 (11.0)	46.9 (14.8)	0.87 (54)	.39
Baseline WEMWBS^f^, mean (SD)		48.1 (5.6)	48.2 (7.2)	0.05 (53)	.96

^a^Presented as chi-square (*df*).

^b^Fisher exact test.

^c^N=29 for unpaid.

^d^DASS: Depression Anxiety Stress Scale.

^e^STAI: State-Trait Anxiety Inventory.

^f^WEMWBS: Warwick-Edinburgh Mental Well-being Scale.

### Engagement

Participants used the app on average 20.9 days (median 22; IQR 7.5) of 4 weeks, completing an average of 28.4 tools (median 25) and completing an average of 8.9 modules (median 7). The mean total duration of use was 164.3 minutes (median 157.0 minutes).

Paid participants used the app on more days (paid: mean days 23.9; unpaid: mean days 18.4; *t*_54_=3.39; *P*=.014; Cohen *d*=0.89), completed more tools (paid: average tools 36.8; unpaid: average tools 21.3; *t*_54_=3.12; *P*=.003; Cohen *d*=0.86), completed more modules (paid: average modules 13.7; unpaid: average modules 4.9, *t*_54_=4.81; *P*<.001; Cohen *d*=1.30), and had a longer total duration of use (paid: 210.2 minutes; unpaid: 125.9 minutes; *t*_54_=3.20; *P*=.003; Cohen *d*=0.88) than unpaid participants.

### Outcomes

**Figure 1:**
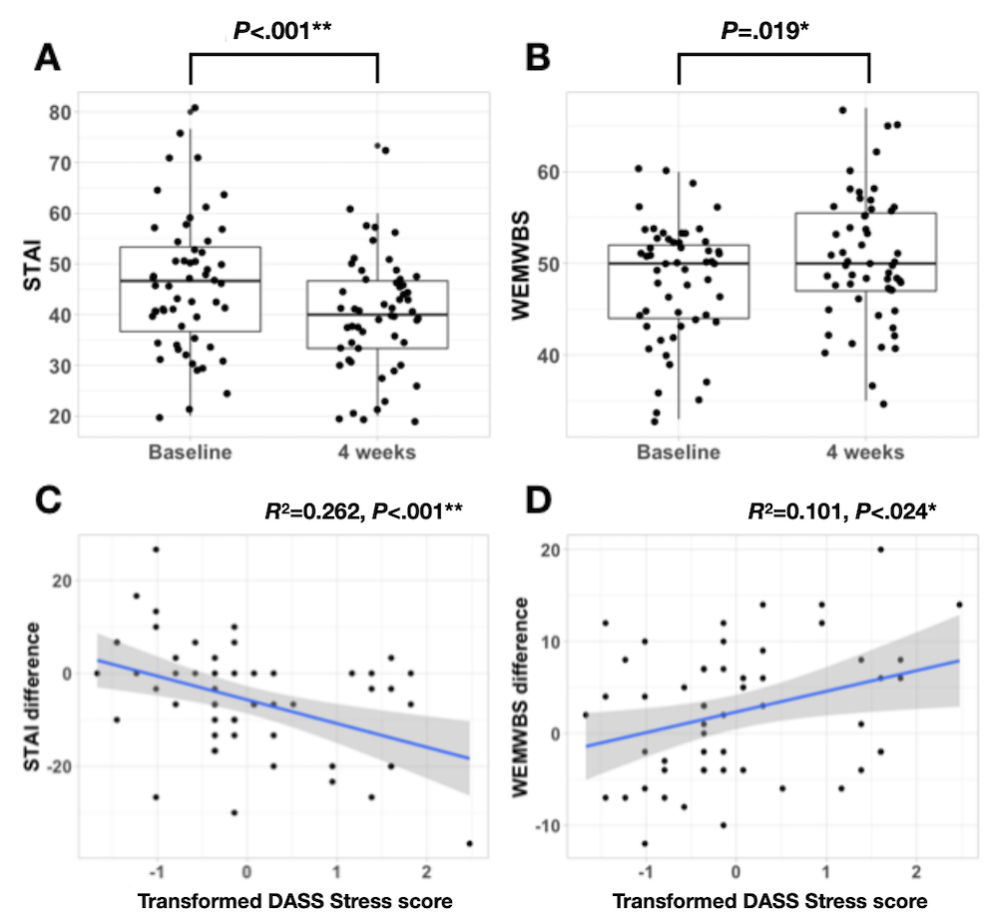
After controlling for paid status, differences in (A) 6-item STAI and (B) WEMWBS after 4 weeks of the BioBase program. Association between transformed DASS Stress scores and difference in (C) STAI and (D) WEMWBS, **P<.01, *P<.05. DASS: Depression Anxiety Stress Scale; STAI: State-Trait Anxiety Inventory; WEMWBS: Warwick-Edinburgh Mental Well-being Scale.

The first 2 hypotheses were supported. After controlling for paid status, linear mixed models revealed that anxiety as measured by STAI significantly decreased (baseline mean 45.52 [SD 13.2]; 4-week mean 39.82 [SD 11.2]; *t*_54_=–3.51; *P*<.001; CI –8.88 to –2.52; Cohen *d*=0.96) and mental well-being significantly increased (baseline mean 48.12 [SD 6.4]; 4-week mean 50.4 [SD 6.9]; *t*_53_=2.41; *P*=.019; CI 0.44-4.23; Cohen *d*=0.66) after 4 weeks of using the BioBase programme. There were nonsignificant effects of paid status on STAI (*t*_54_=1.97; *P*=.054) and WEMWBS (*t*_53_=0.28; *P*=.78; Figure 1).

After controlling for paid status, linear regression models revealed that a higher baseline DASS stress significantly predicted a greater decrease in STAI (*t*_53_=–3.93; *P*<.001; CI –8.62 to –2.80; *R*^2^=0.262) and significantly predicted a greater increase in WEMWBS (*t*_52_=2.32; *P*=.024; CI 0.30-4.12; *R*^2^=0.101; Figure 1).

### Participant Feedback

As much as 32 of 55 participants completed a feedback questionnaire with both multiple-choice questions and free-text answers and were given the opportunity to expand their responses in an interview with an experienced researcher (NH). Responses from 2 participants were withdrawn because the participant did not want their responses shared. Table 2 describes responses from 30 participants (18 paid and 12 unpaid) of the multiple-choice questions.

**Table 2:** Feedback questionnaire results on intervention engagement, usability, and self-reported behavior change.

Theme			Paid (N=18, 60%)	Unpaid (N=12, 40%)	*P* value
**Feasibility and usability**					<.001^a^
	**How many times did you usually log in?**				
		Never, n (%)	0 (0)	0 (0)	
		Once a week, n (%)	0 (0)	1 (83)	
		Several times a week, n (%)	0 (0)	7 (58)	
		Once a day, n (%)	10 (56)	2 (17)	
		Several times a day, n (%)	8 (44)	2 (17)	
	**What time of day did you usually log in?**				.50^a^
		Morning before work, n (%)	3 (17)	2 (17)	
		Morning at work, n (%)	2 (11)	0 (0)	
		Lunch time, n (%)	0 (0)	2 (17)	
		Afternoon at work, n (%)	1 (6)	1 (8)	
		Evening at home, n (%)	12 (67)	7 (58)	
	**Where did you usually log in?**				>.99^a^
		Home, n (%)	14 (78)	10 (83)	
		Work, n (%)	4 (22)	2 (17)	
		Commute, n (%)	0 (0)	0 (0)	
**Acceptability**					
	**Would you recommend the BioBase programme to a friend?**				.13^a^
		Yes, n (%)	15 (83)	6 (50)	
		Maybe, n (%)	3 (17)	5 (42)	
		No, n (%)	0 (0)	1 (8)	
	**Would you like to continue using the BioBase programme?**				.26^a^
		Yes, n (%)	11 (61)	4 (33)	
		No, n (%)	7 (39)	8 (67)	
**Promoting behavior change**					
	**Have there been any changes to your physical health since starting the BioBase programme?**				>.99^a^
		Yes, n (%)	6 (33)	3 (25)	
		Maybe, n (%)	2 (11)	2 (17)	
		No, n (%)	10 (56)	7 (58)	
	**Have there been any changes to your mental health since starting the BioBase programme?**				.06^a^
		Yes, n (%)	5 (28)	3 (25)	
		Maybe, n (%)	3 (17)	7 (58)	
		No, n (%)	9 (50)	2 (17)	
	**Have there been any changes to your engagement at work since starting the BioBase programme?**				.69^a^
		Yes, n (%)	6 (33)	3 (25)	
		Maybe, n (%)	2 (11)	3 (25)	
		No, n (%)	10 (56)	6 (50)	

^a^Fisher exact test.

#### Participant Feedback: Feasibility and Usability

Participants stated that the app was easy to use and navigate (“really good experience” [unpaid participant]). The wearable was also considered usable (“easy to wear and keeps you informed” [paid participant]), with participants paying particular attention to the battery life (“the battery life of the device is exceptional” [paid participant]).

The BioBase programme was convenient and feasible to use as 73% (22/30) of participants reported checking the app at least once per day (“I think we all checked it when we first got in ‘cause we were talking about our sleep and our rubbish sleep, and I guess at lunchtime I’d have a go and then when I’d go home” [unpaid participant]). There was a significant difference between paid and unpaid participants in how often they logged in, with unpaid participants logging in less often (*P*<.001; Table 2).

There were no differences between paid and unpaid participants in the time of day or location in which they logged in; 80% (24/30) of participants logged in at home and 70% (21/30) in the evening or in their lunch break in the workplace (“Yeah normally at home, I do it on my lunch break as well, when I was at work at times, when was stuck in the queue or something” [paid participant]).

#### Participant Feedback: Acceptability in the Workplace

The majority of participants responded they would recommend the programme to a friend (70%, 21/30) and 50% (15/30) would have liked to continue with the programme after 4 weeks. There were no differences between paid and unpaid participants in this respect (Table 2).

BioBase was seen as an acceptable solution in the workplace to support employee health (“I think it’s definitely needed. I think it’s a really great way to track your company satisfaction and well-being and this changes the way people build their HR policies, 100%. I think there are a lot of companies that say they are doing really amazing things, but might not be, and this is a good way to prove it, because you’re not like messing around with the data” [unpaid participant]). Participants appreciated the accessible and personal approach (“on demand, private-ish, lots of great education” [unpaid participant]) and the concept of increasing self-awareness (“it provides great exposure to education and techniques for managing and discussing mental health maintenance,” “The programme has made me more self-aware...I think the BioBase is excellent for workplace use” [unpaid participant]). Many participants had support and encouragement from their colleagues (“I just used the app mostly at work because I was sort of reminded by everyone here” [unpaid participant], “we would do the breathing things, if one of us was stressed” [unpaid participant]).

#### Participant Feedback: Promoting Behavior Change

There were no differences between paid and unpaid participants with regard to perceived changes in physical health, mental health, or engagement at work (Table 2). Key themes in the feedback revealed that real-time data helped participants to understand their current state (“BioBase has helped me become more aware of my body and how external factors were affecting me” [paid participant]) and the impact of environmental triggers (“being aware of what triggers stress and being taught and successful ways of managing this. Has been a game changer” [paid participant]).

Learned proactive and preventative mechanisms from the modules and tools helped achieve positive outcomes (“I approach some tasks, particularly procrastination differently sometimes. I am also even more mindful of taking time to unwind.” [unpaid participant]; “[I learned] how to breathe properly, coping mechanisms for stress, how to get things into proportion where stress concerned and how to cope with stress by developing interests outside work and relaxing after work” [paid participant]). Specifically, the breathing tool was found to be very useful to achieve on the spot stress reduction (“I like the breathing exercises; this is something I will continue with after the trial. I find them very useful for winding down in the evenings before sleep” [paid participant]).

Changes in sleep habits was seen as a consistent behavior change with multiple participants reporting various new health behaviors (“[I] prepare myself for sleep more deliberately than previously” [unpaid participant], “I'm more conscious of going to bed at a reasonable time” [unpaid participant], “I make the room completely dark 30 mins before going to sleep” [paid participant], “Ensuring I go to bed at a particular time even on the weekend” [paid participant]).

There were also reports of increased social connection (“the tribes concept stuck with me and I intend to invest more in building social groups” [unpaid participant], “I consciously try and arrange social activity to increase my activity [levels, like] playing golf, badminton, swimming” [paid participant]).

## Discussion

### Principal Results

This study explores the efficacy of BioBase, which to our knowledge is the first self-guided intervention that combines measurement and biofeedback of passively collected physiological data and linked content on physical and mental health. We found significant decreases in anxiety and increases in mental well-being after 4 weeks of using BioBase in a workplace setting. Importantly, this study found that higher baseline stress levels were associated with greater decreases in anxiety and increases in well-being. These results fit into a wider body of literature showing that digital health interventions may improve symptoms of depression, stress, and anxiety in a workplace environment [35-38], with more pronounced benefit in those with elevated stress levels [10,19].

### Comparison With Prior Work

Self-guided digital health interventions have been shown to have positive effects on mental health and well-being [23] and can be as effective as face-to-face treatments for anxiety [9,39]. The results from this study show BioBase is also potentially effective. The effect sizes found were *d*=0.96 for reduction in anxiety and *d*=0.66 for increase in well-being, which is higher than previous studies using self-guided interventions (*d*=0.44 for work-related worry [9]; *d*=0.47 for anxiety [24]).

The BioBase programme was found to be feasible for use in the work environment, due to ease of access on a personal or work phone [25]. Participants found it relevant, acceptable, and that it worked within their time constraints, as it focused on work-related stressors with simple, short tasks that are easily implemented in the working day [9].

From an employer’s perspective, self-guided interventions do not require the vast resources needed for either traditional organizational support or therapist- or coach-assisted interventions. In addition, they provide more accessibility than in-house training and reach a larger population with comparable clinical outcomes [40]. Our findings suggest that participants with higher levels of stress had significantly greater reductions in anxiety and increases in well-being, which may help employers deliver a solution for those employees most at risk.

Self-guided interventions have the advantages of allowing the user to go at their own pace and access content that is right for them; however, they rely more on an individual’s willingness to take part and often have lower engagement rates or failed adoption [41]. Participants in this study used the app on average 72% of days (20.9/29 days), which is higher than [9,23] or similar to [24] other self-guided digital interventions. It is possible that engagement was higher because the BioBase programme is a unique intervention that provides targeted and relevant content [1] along with integrated technology [25]. The feedback suggests that people appreciated different aspects of the programme based on what content and insights were tailored (ie, feedback on sleep quantity and quality and ability to customize recommended modules).

### Limitations

This study was conducted with participants recruited from a workplace setting across a variety of workplaces and job roles; however, it is beyond the scope of this study to separate participants by type of role or organizational conditions. Future studies with larger samples should be carried out to explore these effects.

The current feasibility study did not utilize a control group and therefore was not able to rule out a regression to the mean or a placebo effect of using a digital intervention. Future studies should conduct a randomized controlled trial to add evidence that the anxiolytic effects were more specific than a placebo effect.

Due to the personalized nature of the intervention and the multiple therapeutic touch points in the BioBase programme, it is difficult to conclude which elements of the BioBase programme contributed most to the observed reductions in anxiety and increases in well-being. Further practical investigation is needed into breaking into the “black box” of digital health interventions to understand the main therapeutic effects [42,43].

## Appendix

Multimedia Appendix 1 Content and theoretical contextualization of the three psychoeducational courses contained in BioBase.

## Abbreviations

DASS: Depression Anxiety Stress Scale
HSE: Health & Safety Executive
STAI: State-Trait Anxiety Inventory
WEMWBS: Warwick-Edinburgh Mental Well-being Scale
